# Ultrassonography at Bedside in Emergency ICU: a powerful diagnostic tool

**DOI:** 10.1186/2036-7902-4-S1-A5

**Published:** 2012-12-18

**Authors:** Herbert Missaka, F Leão, LH Cantarelli, PV Dallava, JEE Almeida, PC Figueiredo, F Nogueira, S Divan-Filho, RS Lannes, FM Vila-Da-Mota, DS Usiglio, G Soriano, LF Jabour, LC Oliveira

**Affiliations:** 1 Intensive Care Unit 2, Hospital Municipal Souza Aguiar, Rio de Janeiro, Brazil; 2 Gama Filho University Medical School, Rio de Janeiro, Brazil; 3 Souza Marques Medical School, Rio de Janeiro, Brazil; 4 Programa de Orientação Científica Medicina, Centro Cultural Tijuca, Rio de Janeiro, Brazil

## Background

Ultrasonography (USG), since the creation at 1954, is contributing as a great diagnostic tool in many medical specialties. Ultrasound has become an integral part of the practice of emergency medicine and trauma care. In this study we report 5 cases, in which USG was fundamental to diagnose and led further decisions in treatment in the Intensive Care Unit.

## Objective

Identify and cases report in which USG was fundamental to diagnose and support the therapeutic choice.

## Patients and methods

Observational prospective study of the patients attended at a public emergency hospital ICU, submitted to the protocol FAST extended (FAST-E), from February until June 2012.

## Results

Five patients were enrolled

Case 1: A 31 year-old woman (ys), with urinary sepsis and mean arterial pressure (MAP)=60 mmHg. USG evidenced hypokinesia of left ventricle, diagnosing cardiogenic shock. Dobutamine was initiated.

Case 2: 34ys man, with severe brain trauma, in mechanical ventilation and O2= 100%, MAP=70 mmHg. USG, at ER, evidenced free fluid in the hepatorenal space, and the surgery was indicated.

Case 3: 50ys woman, with respiratory insufficiency. USG diagnosed an hypertensive pneumothorax. Drainage was perfomed.

Case 4: 66ys man, victim of a spinal trauma, MAP=75 mmHg and inferior vena cava diameter=28 mm, collapsibility <50%. FAST-E protocol evidenced neurogenic shock. Norepinephrine was initiated.

Case 5: 52ys woman, related subclavian vein thrombosis treatment 2 months ago, and was referred to ER with brawny edema of the and arms. USG showed a superior vena cava thrombus and absence of line A in the left pulmonary apex, featuring superior vena cava syndrome (thoracic CT, after USG, demonstrated pulmonary artery e superior vena cava thrombus and occlusive apex tumor). Anticoagulation with LWMH was initiated.

## Conclusion

The reported cases with severe diseases were diagnosed by USG examination at the ICU. The incorporation of this technology, as a routine in the ICU, demonstrated efficacy, empowered diagnostic decisions, and allowed reliably treatment.
